# Variation of the 3’RR1 HS1.2 Enhancer and Its Genomic Context

**DOI:** 10.3390/genes15070856

**Published:** 2024-06-29

**Authors:** Carla Jodice, Patrizia Malaspina, Bianca Maria Ciminelli, Cristina Martinez-Labarga, Michela Biancolella, Giuseppe Novelli, Andrea Novelletto

**Affiliations:** 1 Department of Biology, University of Rome Tor Vergata, 00133 Rome, Italy; patrizia.malaspina@uniroma2.it (P.M.); bianca.ciminelli@uniroma2.it (B.M.C.); martine@uniroma2.it (C.M.-L.); michela.biancolella@uniroma2.it (M.B.);; 2 Department of Biomedicine and Prevention, University of Rome Tor Vergata, Tor Vergata University Hospital, 00133 Rome, Italy; novelli@med.uniroma2.it

**Keywords:** HS1.2 Ig enhancer, Ig allotypes, immunoglobulins, transcription factor binding

## Abstract

In humans, the HS1.2 enhancer in the Ig heavy-chain locus is modular, with length polymorphism. Previous studies have shown the following features for this variation: (i) strong population structuring; (ii) association with autoimmune diseases; and (iii) association with developmental changes in Ig expression. The HS1.2 region could then be considered as a contributor to inter-individual diversity in humoral response in adaptive immunity. We experimentally determined the HS1.2-length class genotype in 72 of the 1000 Genomes CEU cell lines and assigned the HS1.2 alleles to haplotypes defined by 18 landmark SNPs. We also sequenced the variable portion and ~200 bp of the flanking DNA of 34 HS1.2 alleles. Furthermore, we computationally explored the ability of different allelic arrangements to bind transcription factors. Non-random association between HS1.2 and Gm allotypes in the European population clearly emerged. We show a wealth of variation in the modular composition of HS1.2, with five SNPs further contributing to diversity. Longer alleles offer more potential sites for binding but, for same-length alleles, SNP variation creates/destroys potential binding sites. Altogether, the arrangements of modules and SNP alleles both inside and outside HS1.2 denote an organization of diversity far from randomness. In the context of the strong divergence of human populations for this genomic region and the reported disease associations, our results suggest that selective forces shaped the pattern of its diversity.

## 1. Introduction

The human locus containing the coding segments for the immunoglobulin heavy-chain constant domains is a result of tandem duplication, in which the telomeric member harbors, among others, the active gene segments IGHG3, IGHG1, and IGHA1, whereas the other member contains IGHG2, IGHG4, IGHE, and IGHA2. All these gene segments encode for the heavy-chain constant domains of different immunoglobulin classes. They are transcribed from telomere to centromere on the minus (−) DNA strand. A regulatory region (3′RR) is present in each of the duplicated blocks, with the telomeric and centromeric paralogs named 3’RR1 and 3’RR2, respectively. In humans, each of these latter regions contains three enhancer elements, indicated as HS3, HS1.2, and HS4 (Figure 1 in refs. [1,2]). Both regions have been categorized as super-enhancers, i.e., elements that drive expression of genes that control and define cell identity. Typically, super-enhancers are formed by multiple elements, each capable of binding transcription factors synergistically [3]. 

Several aspects of the functioning and role of the 3′RR have been investigated in mice (reviewed in ref. [4]), in which a single paralog is present. Moreover, in mice, the entire region includes inverted repeat sequences flanking HS1.2, which diverge from humans in their internal sequence [5]. In a multi-species comparison, it seems that evolution maintained a ‘quasi-palindromic’ organization, at least in mammals, making it tempting to speculate that this unique arrangement is a structural feature of this regulatory region [6]. Evidence accumulated by studying several knock-out mouse models shows that the 3′RR portion including the quasi-palindrome dictates antigen-dependent locus remodeling (global somatic hypermutation and class switch recombination to major isotypes) in activated B cells, and antibody production in plasma cells [7].

In humans, the 3′RR1 HS1.2 is itself modular, with length polymorphism resulting from variation in the number of internal modules across gene copies. Traditionally, four size classes (named 1 to 4 from smallest to largest) can be identified through PCR and gel electrophoresis. Each class differs by approximately 60 bp, on a total length determined by the particular amplification protocol used [8,9]. Previous studies have shown the following features for this variation: (i) strong population structuring; (ii) association with autoimmune diseases; and (iii) association with developmental changes in Ig expression [10,11,12,13,14,15,16,17]. Conversely, the 3′RR2 HS1.2 displays low polymorphism, with only two alleles in the European population, the major of which accounts for 93% [9].

Due to these features, the HS1.2 region could be considered as a contributor to inter-individual diversity in humoral response in adaptive immunity. By resequencing the region immediately telomeric to HS1.2 from allele 1 and 2 in homozygous individuals, a strong non-random association (linkage disequilibrium, LD) between HS1.2 and four neighboring SNPs (rs12896746-rs12896897-rs7144089-rs7143677) was detected [18]. In these authors’ data, these SNPs are arranged in virtually two haplotypes only (A-C-G-A and G-T-C-G), that become predictors of the HS1.2 allele in cis. By leveraging this information, we inferentially suggested that in diverse human populations, some degree of disequilibrium extends all the way from HS1.2 to IGHA1, IGHG1, and IGHG3, the latter lying 60 kb apart [19]. In this context, it is worth noting that variation in IGHG1 and IGHG3 includes missense variants long shown to be responsible for the immunogenic determinants of Gm allotypes [20].

In this study, we used a more direct experimental approach to investigate linkage disequilibrium in the entire genomic region surrounding HS1.2, spanning from 100 kb centromerically to 60 kb telomerically of it. We experimentally determined the HS1.2-length class genotype in 72 of the cell lines (Table S1) that were fully sequenced for CEPH European Utah residents with Northern and Western European ancestry (CEU) subset of the 1000 Genomes project [21] and assigned the HS1.2-length alleles to haplotypes defined by 18 landmark SNPs. Among these, 11 correspond to missense variants, i.e., 2, 2, 4, and 3 in IGHA2, IGHA1, IGHG1, and IGHG3, respectively, 7 of which determine allotypic diversity in IGHG1 and IGHG3 and the ensuing changes in immunogenicity [22]. Furthermore, a relevant role of this set of variants on the biology of immunoglobulin genes derives from the differential association of their genotypes with expression level or splicing of IGHC gene segments, thus qualifying them as eQTL and/or sQTL [23]. These effects are often tissue-specific and disease-related. For example, a signal of association with the intrathecal synthesis of IgG was found for SNPs around IGHG3 in multiple sclerosis [24]. 

In this study, we aimed at providing a clearer picture of the non-random association between HS1.2, the adjacent non-coding SNPs, and the more distant Gm allotypes in a cohort of subjects of European ancestry.

During the development of this work, we also noticed subtle variation within HS1.2 length classes, that was detectable upon agarose gel electrophoresis but not clearly sizeable. As the diversity of HS1.2 cannot be safely captured by high throughput methodologies with short reads, due to its modular nature, we Sanger sequenced amplicons including the variable portion of 34 HS1.2 alleles and ~200 bp of flanking DNA from homozygous individuals or after cloning and recombinant selection.

We show a wealth of variation that could not be detected with the separation methods used so far and predicts a much larger repertoire with larger screenings.

Multiple lines of evidence denote that the 3′RR region harbors variation contributing to the qualitative and quantitative transcriptional output of IGH gene segments. First, flag SNPs showing association with serum Ig class ratios have been identified in the region [25]. Second, variable mRNA expression in lymphoblastoid cell lines, depending on the HS1.2-inferred genotype, was reported [19]. Third, an estrogen-dependent sex-biased Chip-seq enrichment was observed in the mouse [26,27] and was also paralleled by an excess of estrogen-binding sites in humans [28]. Interestingly, a sex-biased response was also reported for clinical implications in COVID-19 [16].

Experimental data support the ability of super-enhancers to bind an array of transcription factors more than the sum of their internal elements [3]. Binding sites for transcription factors have repeatedly been reported for HS1.2 [6,8,9,29]. The new sequence diversity here reported prompted us to explore computationally the allele-specific content in putative transcription factor binding sites. In doing this, we wanted to explore the role of changes in the modular composition of HS1.2-length alleles and internal SNPs by analyzing the length-invariant CORE, END, and length-variable (VAR) sub-portions separately.

## 2. Materials and Methods

### 2.1. HS1.2-Length Typing

The list of the 72 cell line DNAs used is reported in Table S1. For both lymphoblastoid cell and donor blood DNA, fragments containing the VNTR region of HS1.2 were PCR-amplified according to [8], with minor modifications. In particular, the reactions were carried out in 30 µL of a mix made up of 200 ng genomic DNA, 0.2 mM of each dNTP, 0.1 mM of each primer, 1× Taq Platinum plus buffer and 0.5 U Taq Polymerase (Platinum plus, Invitrogen^TM^, Thermo Fisher Scientific Inc., Waltham, MA, USA). PCR was performed on a Step One (Applied Biosystem^®^, Thermo Fisher Scientific Inc., Waltham, MA, USA) thermal cycler. Denaturation was at 94 °C/3 min, followed by 10 cycles consisting of 94 °C/45 s, 65 °C/55 s, and 72 °C/45 s, and 20 additional cycles with annealing at 63 °C/105 s. A final elongation step was carried out at 72 °C for 10 min. The PCR products were then analyzed on 1% agarose gel stained with ethidium bromide, and the alleles were classified according to the length of the PCR fragments (allele 1, ca. 300 bp; allele 2, ca. 350 bp; allele 3, ca. 400 bp; allele 4, ca. 450 bp). 

### 2.2. Handling of the 1000 Genomes Data

Individual genotypes at each of the 18 SNPs listed in Table S1 were obtained with the data slicer available at http://www.ensembl.org/Homo_sapiens/Tools/DataSlicer (accessed on 2 February 2024). 

Genotypes at rs61984162 and rs12433324 in cell lines NA10839, NA12752, NA12003, and NA12145 were experimentally validated with TaqMan assays (Thermo Fisher Scientific^TM^, Waltham, MA, USA) n. C__90305355_10 and C__26668621_10, according to the manufacturer’s instructions. 

The whole 72 × 18 dataset was handled in a spreadsheet to insert the experimental results of HS1.2 typing (Table S1) and to produce the Phase2 [30] input file. Missing genotypes at rs1045853 (3) and rs77307099 (10) were replaced with ??, as per the instruction manual. The program was run with an elongated chain (1000, 10, and 1000 for the number of iterations, thinning interval, and burn-in, respectively).

In order to project the population coverage of the haplotypes identified here, we inferred the occurrence of the haplotypes defined by the same 18 SNPs in the full series of 503 subjects of European descent (EUR) of the 1000 Genomes project, retaining the original phasing [21].

Measures of LD and the visualization of haplotype blocks were obtained with Haploview [31]. As the implemented methods only allow for strictly biallelic markers, only homozygotes and heterozygotes for HS1.2-length alleles 1 and 2 were included (as reported in Table S1).

### 2.3. DNA Sequencing

We selected cell line/individual DNAs to undergo HS1.2 sequencing according to the following criteria: (1) putative homozygotes showing a single sharp band on agarose gel; (2) apparent homozygotes for length alleles departing from the canonical sizes reported above (single puffy band); and (3) heterozygotes for large (3,4) alleles uncommon among CEUs. Subsequently, 2 ng/100 bp of PCR products from homozygous individuals were dried for 40 min, at 65 °C and cleaned up with exonuclease I and shrimp alkaline phosphatase, according to standard protocols. Sequencing was performed on both strands with each of the same primers used in the original PCR. 

In case (1), the electropherogram was clearly readable, whereas in cases (2) and (3), the electropherogram was compatible with the overlap of two sequences differing in base composition and/or length. In these latter cases, to determine the HS1.2 sequence, PCR amplicons were cloned into pGEM^®^-T Easy Vector (Promega, Madison, WI, USA) to transform DH5α *Escherichia coli* competent cells. Plasmid DNA from recombinant clones was purified by standard techniques and sequenced on both strands using flanking plasmid-specific primers. 

### 2.4. Search for Putative Transcription Factor Binding Sites and Other Elements

The sequence of each HS1.2 allele was converted to fasta format and analyzed with TFBIND (https://tfbind.hgc.jp, accessed on 2 February 2024) [32] to find putative transcription factor binding sites. This tool uses a weight matrix in the transcription factor database TRANSFAC [33], and originally estimated cut-offs. The analysis was performed with the complete sequence of each allele, and the results partitioned for the CORE (first 94 positions), VAR (variable), and END (last 83 positions) portions of each allele. This was obtained by handling the results of each run in a spreadsheet to compile a direct comparison between outputs. Notably, the assignment of a putative transcription factor binding site to each of the regions is based on the position of the first nucleotide of the binding motif, and some motifs may overlap the boundaries between two regions.

## 3. Results

### 3.1. HS1.2-Length Allele Contributions to Haplotype Blocks 

**Figure 1:**
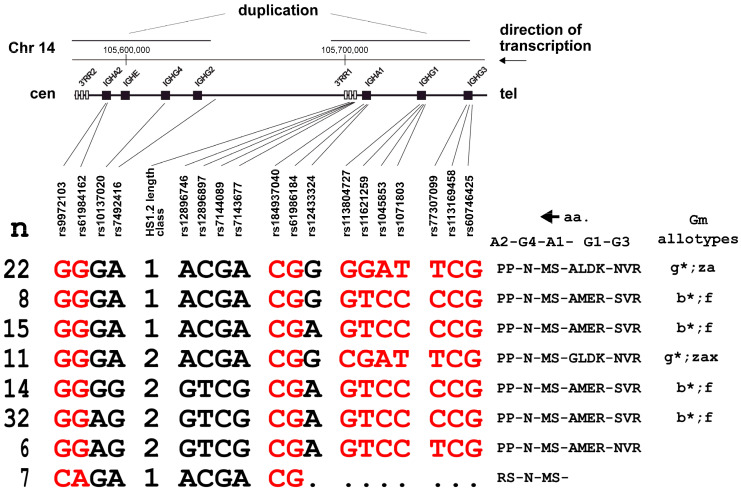
The 8 most common 19-loci haplotypes reconstructed from 72 cell lines. A simplified genomic map is reported at the top, showing the genomic position of each marker. Below, from left to right: absolute number of inferred haplotypes, haplotype composition with missense SNP alleles in red (amino acid substitutions listed in Table S1), the corresponding amino acid in the heavy-chain constant region (ordered from right to left), the corresponding Gm allotype with the classical nomenclature reconstructed from https://www.imgt.org/IMGTrepertoire/Proteins/allotypes/human/IGH/IGHC/G1m_allotypes.html (accessed on 2 February 2024). The 29 remaining haplotypes are listed in Table S2, top, and Figure S1.

Interpreting the HS1.2 and 18 SNP allele arrangements in the 144 chromosomes of the 72 cell lines resulted in 28 different haplotypes (Table S2). Of these, 12 were singletons, whereas 16 had frequencies ranging from 2 to 32. The 7 most common haplotypes accounted for 75% of the 144 total haplotypes (Figure 1, rows 1–7), denoting strong non-independent arrangements of marker alleles. 

HS1.2 allele 1 is clearly preferentially associated with the G-G-G-A and A-C-G-A sub-haplotypes located centromerically and telomerically to it, respectively. On the other hand, HS1.2 allele 2 is preferentially associated with the G-G-A-G and G-T-C-G sub-haplotypes at the same SNP loci. 

The uncommon rs61984162 allele A in IGHA2 (Figure 1, last row) was found on four different haplotypes, three of which (Id 25, 26, and 27 in Table S2) carried the HS1.2 allele 1 (seven allele copies). 

By inspecting the array of haplotypes in more detail, we identified six broader families (Table S2 and Figure S1), which accounted for 129 out of 144 haplotypes (89.6%). Two such families include the vast majority of HS1.2 allele 1 linked to the G-G-G-A sub-haplotype centromerically and extending to IGHA1 telomerically (orange and light blue). One family included HS1.2 allele 2 linked to the G-G-G-A sub-haplotype centromerically and extended up to IGHG1 telomerically (pink). A medium-frequency family included HS1.2 allele 2, linked only to a centromeric G-G-G-G block (green). A high-frequency family included HS1.2 allele 2 linked to the G-G-A-G sub-haplotype centromerically and extending to IGHA1 telomerically (light green). Finally, one family included HS1.2 allele 1 linked to the C-A-G-A sub-haplotype centromerically, as mentioned above, and extended to IGHA1 telomerically (ochre). Four of the five HS1.2 alleles 3 and 4 were found on two haplotypes, sharing the centromeric G-G-G-A sub-haplotype (no color).

The frequencies in our limited series were similar to the larger series of 503 Europeans (Table S2, bottom), with the seven most common haplotypes accounting for 63.5%. It is worth noting that relevant discrepancies were observed among the five European subpopulations, in line with the strong structuring of the IGH locus [21]. These patterns denote a fairly strong degree of LD across the entire IGH locus, which apparently strongly links HS1.2 and markers at a distance up to 100 kb on the centromeric side and at least 30 kb on the telomeric side.

We used Haploview to quantify LD and visualize haplotype blocks (Figure S2).

On the centromeric side, strong disequilibrium was confirmed between HS1.2 and two SNPs in IGHG4 and IGHG2 at approximately90 kb. On the telomeric side, four markers displayed almost complete disequilibrium, confirming the initial findings [18]. Further telomerically, the disequilibrium of HS1.2 decays abruptly in the interval rs7143677–rs61986184 and decreases with distance. Conversely, disequilibrium between SNPs remains strong all the way to rs1071803 in IGHG1.

### 3.2. Internal Sequence Heterogeneity of HS1.2 Alleles

Separation of HS1.2 alleles on agarose gel electrophoresis revealed subtle variation within length classes, which was not clearly sizeable. A review of the images reported in previous papers confirmed this observation, which has already prompted detailed investigations [9]. We then engaged in the characterization of this level of variation by directly sequencing a number of allele copies, obtained from apparently homozygous 1/1 or 2/2 DNA samples or from carriers of alleles 3 and 4, unusual in CEUs, upon cloning. Sequencing covered positions homologous to 14:105696578-105696260 (GRCh38/hg38), as numbered in the RefSeq NT_026437.11. 

**Figure 2:**
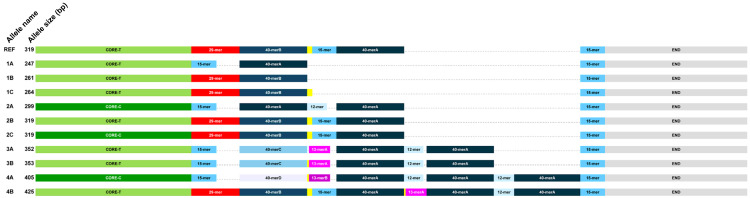
Internal structure of HS1.2 alleles characterized in this study.

We found 10 different allele structures, graphically represented in Figure 2 and further detailed in Tables S3–S5. Over the surveyed DNA, three main regions were observed, i.e., a “CORE”, invariant in length, spanning pos. 14:105696578-105696485; a portion variable in length spanning positions equivalent to 14:105696484-105696343 in RefSeq; and an “END” portion, invariant in length, spanning pos. 14:105696342-105696260. The overall allele lengths fell into the following classes: 247 to 264 bp, grouped into allele 1; 299 to 319 bp, grouped into allele 2; 352 and 353, for allele 3; and 405 to 425, for allele 4.

The structure of the variable portion was clearly modular, with different module compositions within each length class (Table S3). The modules are listed in Table S4. In particular, as observed previously [8], the main contributor to length differences was a 40 bp module (40-mer) which was found to be repeated one to four times in alleles 1 to 4, respectively. In between the CORE and the first 40-mer (in the order shown in Figure 2 and Table S3), either a 29-mer or a 15-mer could be found in alleles 1, 2, and 4, whereas among the two copies of allele 3, only the 15-mer was found. These two alternative modules strongly differ in their base composition, with the 15-mer having a G-C content of 93% as opposed to 76% in the 29-mer. In between the first and second 40-mer copy (alleles 2–4), a 12-, 13-, or 15-mer can be found, all of which are very G-C-rich. These can be accompanied by a C_1–3_ stretch. A similar organization is present between the second and third 40-mer (alleles 3,4). Finally, in the two copies of allele 4, only the 12-mer was found between the third and fourth 40-mer. In all sequenced alleles, a copy of the 15-mer is invariably present at the boundary with the END segment.

### 3.3. Single Nucleotide Variation in the CORE and the Modules

A variable position was found in the CORE, identified as rs373084296. We distinguished copies carrying each of the two SNP alleles as CORE-C and CORE-T, respectively (Figure 2 and Table S3).

Within the 40-mer copies, we found four variable positions, with four arrangements in cis (Table S4 top). The four different 40-mers were thus distinguished in 40-mer A to D. It is worth noting that only the 40-mer closest to the CORE was found to vary. This copy of 40-merA was found adjacent to the 15-mer in alleles 1 and 2 only. The 40-merB was found adjacent only to the 29-mer in alleles 1, 2, and 4. The single 40-mers C and D were adjacent to the 15-mer. The second to fourth additional copies of the 40-mer in alleles 2–4 were invariably of the 40-merA type. No variation was found in the END segment.

In summary, among 34 sequenced alleles, we found three, three, two, and two different types for class 1, 2, 3, and 4, respectively (named in Tables S3 and S5, leftmost column). Overall, we sequenced 14 allele 1s (Table S5), 12 of which turned out to be of the 1A type, and 16 allele 2s, 8 of type 2A and 7 of type 2B. Allele 2B is identical to RefSeq in the DNA region surveyed. We note here that the relative proportions of the different types do not represent population frequencies, due to the strong selection of candidates to sequencing.

### 3.4. Putative Impact of HS1.2 Diversity on Transcription Factor Binding Sites and Other Elements

We computationally explored the ability of HS1.2 to bind transcription factors, by analyzing the CORE, the length-variable portion (VAR) and the END, separately (Table S6). We focus here on a subset of transcription factors motifs repeatedly reported in the literature.

The transcription factor motif for Oct1 was present in the CORE equally in all alleles described here, as an octanucleotide invariant across our sequences, beginning at 14:105696565, confirming previous data [9].

Motifs for SP1 were found in multiple positions, i.e., the CCCGCCCCCT(C) motif of the 15-mer, e.g., at 14:105696412 [6,34], and the GGGACACCC motif at the boundary between the 40-mer and the 12-, 13-, or 15-mer, e.g., 14:105696363 [8]. As these motifs are increasingly repeated along the allele 1–4 series, the number of predicted binding sites increases accordingly (Table S6). Interestingly, motifs for SP1 are not present in the 29-mer, reducing the number of sites in allele 1A as compared to 1B and 1C. 

Conversely, the number motifs for NFkB, which is predicted to bind the boundary between the 40-mer and the 15-mer, increase regularly with allele size. 

A binding motif for AP1 (11 bp) starts at 14:105696444 in the 40-mer and covers a variable position. The 40-merB has a C at rs78955324, which reduces the match to the consensus to 10/11. 

AP4 and MyoD have similar binding motifs, which are found in the 29-mer (14:105696482) and at the boundary between the 15-mer and the 40-mer (14:105696405). This situation somewhat compensates between alleles with and without the 29-mer, with the opportunity to bind AP4 (Table S6).

Stat3 was reported to bind super-enhancers more than typical enhancers [3]. A site compatible with the consensus motif is invariably present in the CORE at 14:105696515. A second site is found at the boundary of 40-mer (14:105696431) when followed by C_5_. Thus, alleles 1A and 1B lack this second site.

In addition to transcription factor binding motifs, other regulatory elements have been identified in HS1.2. Moreover, these elements may be affected by both sequence and structural variation. A DRE (Dioxin Response Enhancer) GCGTG motif [34] was found to start at 14:105696454. However, we predict that this motif is disrupted in the 40-merB, which carries the reference allele T at rs28624614. Therefore, alleles 1B and 1C lack this element completely, whereas other alleles have one or more copies in their 40-merA specimens.

The mouse hs1.2 homologue showed a peak of estrogen receptor binding [26,27]. A 13 bp stretch beginning at 14:105696450 in the 40-merB bears a 12/13 match with the Estrogen receptor Response Elements (ERE) consensus [16,28]. This sequence overlaps two of the variable positions detected here. For the first (14:105696448), both alternative alleles match the ERE consensus, but for the second (rs78955324), the alternative allele T does not. Thus, in this case, the cumulative ability of HS1.2 to bind may also depend on the number of 40-mer repeats and on their particular types.

## 4. Discussion

This study was inspired by our previous observation [19] that largely divergent 3′RR1 HS1.2 allele frequencies across global populations parallel diverse IgH allotype frequencies [35]. We thus worked out the non-random arrangement of HS1.2 and neighboring SNP alleles in the Ig heavy-chain coding segments on chromosome 14, by directly testing cell lines from subjects of European descent. In phasing our experimental results with genotype calls obtained by whole genome resequencing, we considered only a subset of SNPs enriched in missense and regulatory variants, which impact the functional and immunogenic properties of Ig heavy chains. While some of these did not display polymorphism among the 72 CEUs examined here, they are nevertheless variable in other continental backgrounds (African and East Asia), contributing to the global differentiation of this genomic region. 

Our results confirm the strong disequilibrium between HS1.2-length alleles 1 and 2 and the four flanking SNPs of rs12896746, rs12896897, rs7144089, and rs7143677 [18]. However, we show that non-independent arrangements also involve the more distant IGHG4 and IGHA2 gene segments on the centromeric side and, to some extent, the IGHA1, IGHG1, and IGHG3 s well on the telomeric side. The particular haplotypic combination reported here replicates those inferentially predicted [19]. In a recent report, a larger number of haplotypic combinations at the same four SNPs was reported [16]. Our reconstructions did not confirm three of them. Among haplotypes confirmed in both studies, the A-C-G-A and the G-T-C-G arrangements in cis were associated preferentially with HS1.2 alleles 1 and 2, respectively.

Overall, the disequilibria between SNPs appear to be stronger than between SNPs and HS1.2, an indication that the latter accrues diversity at a faster pace, but not enough to reach randomization. In fact, it has been hypothesized that in HS1.2 of both rodents and humans, the generation of multiple alleles has likely been favored by its central position within a large palindromic region [29].

We stress that the above results were obtained on the CEUs of the 1000 Genomes Project [21]. In the context of the strong divergence of human populations in this genomic region [18,19], they call for validation in populations with continental ancestry other than Europeans. 

Our sequencing results reveal a repertoire of HS1.2 alleles more variegated than previously described [1,6] and documented in deposited accessions. Both structural and single nucleotide variation contribute to this heterogeneity. Allelic variants of the length-variable region differ in the number and type of modules. In some cases, this diversity implies remarkable variation in the G-C content. Single nucleotide variation at several positions further adds to diversity. The finding of 10 haplotypic arrangements among 34 sequenced alleles predicts that an array of additional variants may be found upon larger screenings. Additionally, it is to be expected that populations in which alleles 3 and 4 are prevalent may display even larger repertoires, due to the higher propensity of repeated segments to undergo unequal exchanges.

Our computational search showed that the occurrence of motifs for binding transcription factors or other regulatory effectors is not simply proportional to allele length, due to two factors: (i) the particular modules contributing to an allele of a given length class; and (ii) the particular module type, especially for the 40-mer. 

Overall, the results reported here add support to the concept that the investigated genomic region contributes to inter-individual variation in IgH output through at least three mechanisms: (i) the regulatory activity of HS1.2 during B-cell maturation and the isotype preferentially produced; (ii) the quantitative IgH output; and (iii) specific properties of the particular allotypes. While the joint action of these three factors might be the basis of the observed disease associations of HS1.2, it is indeed possible that some subtypes within a length class contribute disproportionately. The notion that LD spans the entire IGH locus [22] on one side complicates the interpretation of functional consequences of individual variants, but allows for potential synergy between variants in conferring peculiar properties to the final product(s) of specific haplotypic arrangements. This may call for a new generation of studies in which targeted typing could eventually reveal association to a specific allele sub-type.

In conclusion, the current results require a new wave of studies on the specific properties of each variant, both in vivo and in vitro. In this context, previously reported disease associations [11,13,14,16,17] and the degrees of variation identified here align with the observation that sequence variation occurs in transcription regulatory regions of the genome, primarily enhancers, and variation has a disproportionate impact on super-enhancer domains [3]. Finally, the identification of several haplotypes potentially associated with human diseases can serve as a basis to generate studies of molecular genetics with associated immunological factors in the susceptibility to human infectious diseases. 

## Data Availability

The original data presented in the study are openly available in GenBank (Acc. n. PP841115-PP841124).

## Supplementary Materials

The following supporting information can be downloaded at: https://www.mdpi.com/article/10.3390/genes15070856/s1; six supplementary tables cited in the text are in a single Excel spreadsheet. Table S1: List of SNP loci flanking HS1.2 and considered in the analysis of linkage disequilibrium, experimentally typed cell lines of the 1000 Genomes project and subset included in Haploview display; Table S2, top: Phased 18-SNP haplotypes encompassing HS1.2 in 72 CEU cell lines experimentally typed; bottom: Frequencies of haplotypes defined by the same 18 SNP in the 503 EUR subjects of the 1000 Genomes project, and 5 subpopulations; Table S3: Internal structure of HS1.2 alleles characterized in this study. The sequence on the (−) strand is shown. Variable positions are shown in red. Positions are numbered according to GRCh38/hg38. Landmark positions discussed in the text are reported in the top row; Table S4: Internal sequences of HS1.2 sub-modules. The sequence on the (−) strand is shown. Variable positions are shown in red; Table S5: Distribution of the 34 resequenced alleles by different internal structure and length class; Table S6: Summary results of the search of putative transcription binding sites with TFBIND. Each entry reports the number of identified motifs with the starting base falling in the reported portion of HS1.2; Figure S1: Same data as Table S2 (top), with color shading of similar haplotypes as discussed in the text; Figure S2: Plot of linkage disequilibrium colored according to D’ in phased chromosomes carrying HS1.2 alleles 1 or 2. The distance between markers is reported to scale. LD values for the two rightmost markers are not reported as these are monomorphic in this smaller series.
