# Supplementary figures and images for: Variation of the 3’RR1 HS1.2 Enhancer and Its Genomic Context
Source: Genes (Basel). 2024 Jun 29;15(7):856. doi: 10.3390/genes15070856 (PMC11275349; doi:10.3390/genes15070856)

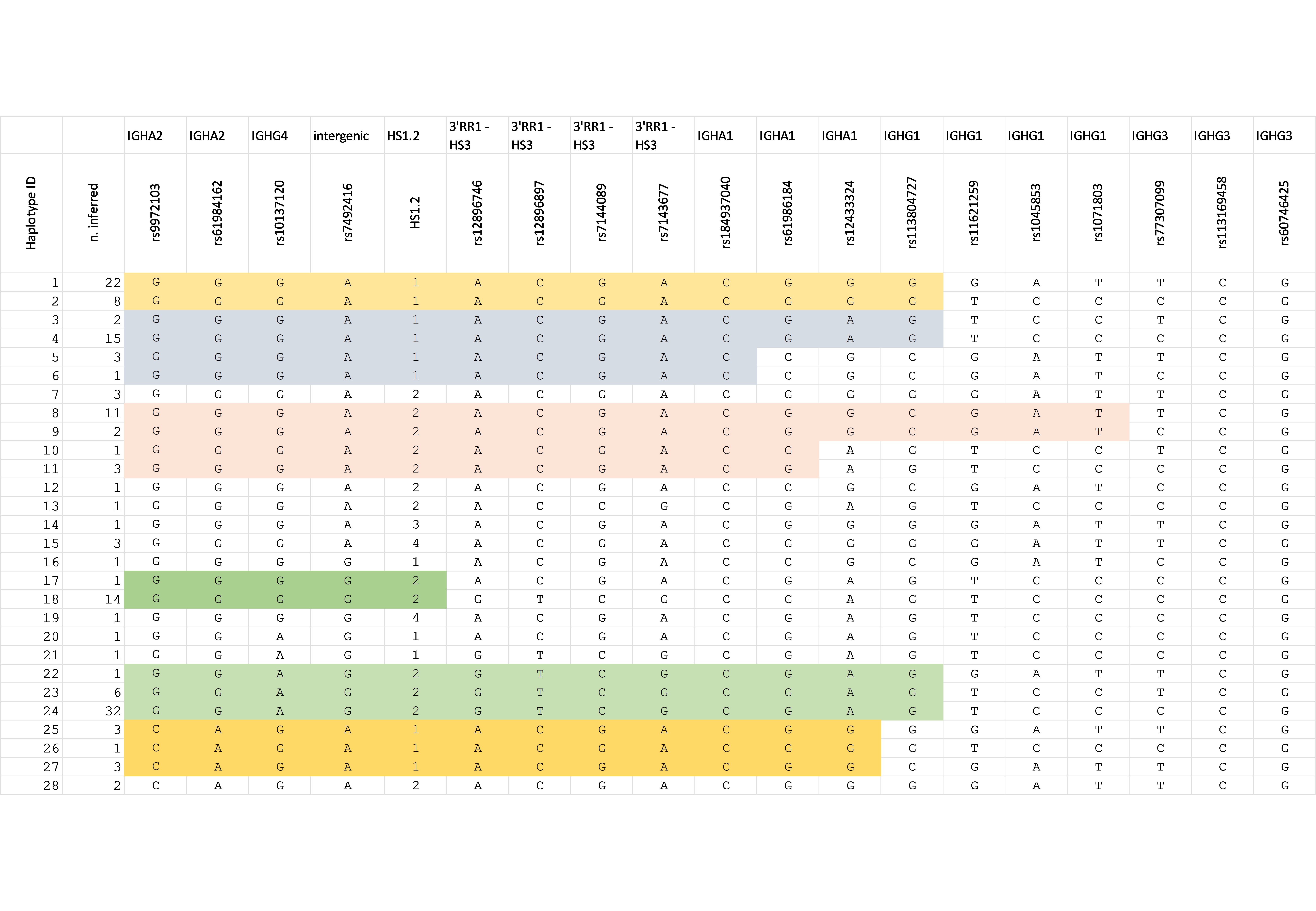

Supplement: Supplementary file 1 [file genes-15-00856-s001.zip › SupplFigS1.jpg]

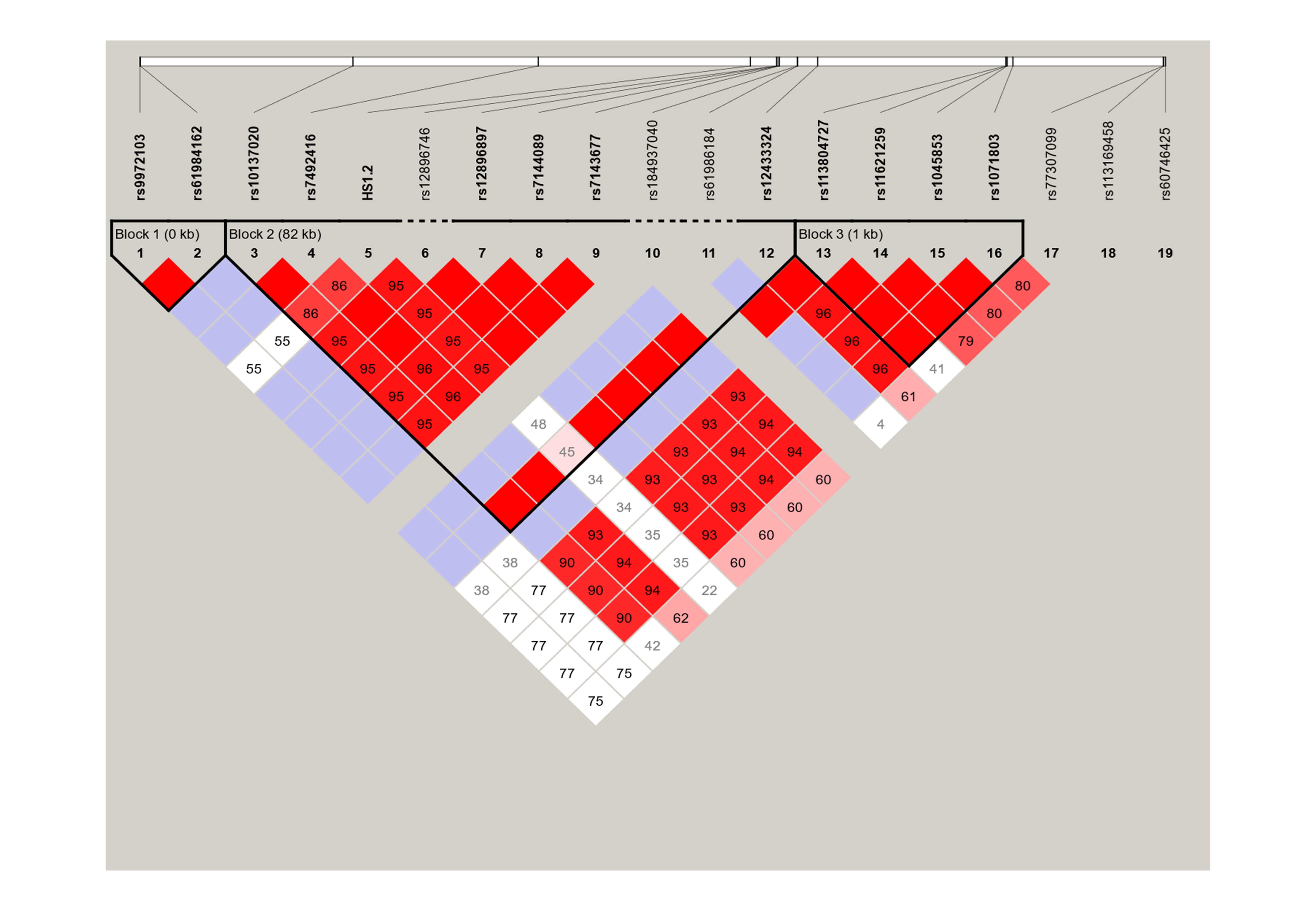

Supplement: Supplementary file 1 [file genes-15-00856-s001.zip › SupplFigS2.jpg]
